# Primary Care Provider Attitudes and Perceptions of Routine Cognitive Screening in Older Adults

**DOI:** 10.1093/geroni/igaa057.3257

**Published:** 2020-12-16

**Authors:** Giovanna Potenza, Meghan Mattos

**Affiliations:** University of Virginia, Charlottesville, Virginia, United States

## Abstract

The 2011 Annual Wellness Visit (AWV) Medicare benefit includes a cognitive screening component intended to improve screening of older adults. However, available literature only presents physician perspectives on cognitive screening prior to 2011. The purpose of this study was to explore primary care provider (PCP) attitudes and perceptions of cognitive screening in older adults. An Internet-based survey link was sent to Virginia professional organizations and clinics to distribute to PCPs serving older adults. Likert scale, multiple choice, and free response questions were used to understand current attitudes, perceptions, and practices. The sample (N=39) was comprised of 26 nurse practitioners (NPs), 9 physicians, and four who did not disclose role. Most participants were aware of the AWV (n=31, 88.6%) and agreed that early detection “promotes earlier diagnosis and access to resources” (mean ± standard deviation,1.58±0.69). However, less than half of NPs screened annually (n=10/26, 38.5%) and even less conducted screening during an AWV (n=7/26, 26.9%). About half of MDs conducted cognitive screening during an AWV (n=5/9, 55.6%). Although NPs screened less, they more strongly agreed that screening should occur annually (1.92±1.15 vs. 2.67±1.23) and “additional training would improve [screening] ability” (2.04±1.0 vs. 3.22±1.20). Also, few NPs independently managed impairment (n=5/26, 19.2%) compared to MDs (n=5/9, 55.6%). Our findings demonstrate that NPs screen less and feel less prepared to conduct cognitive screenings. It is important to provide additional resources and training for all PCPs, but especially NPs who are rapidly entering primary care to help improve identification and management of cognitive impairment.

